# Supplementary material for: Effects of bear endozoochory on germination and dispersal of huckleberry in the Canadian Rocky Mountains
Source: PLoS One. 2024 Nov 6;19(11):e0311809. doi: 10.1371/journal.pone.0311809 (PMC11540177; doi:10.1371/journal.pone.0311809)
Supplement: S1 Appendix — (DOCX) [file pone.0311809.s001.docx]

**Appendix I**

**Table A1:** Bear Scat and huckleberry sample collection data from Waterton Lakes National Park, AB and the Elk Valley, BC. Data collected includes collection number, collection location, collector, collection date and scat freshness (if applicable). All samples were collected during the field season of 2021.

**Table A2:** Germintation trial data per treatment group (Whole Berry; Seeds from Berry; Seeds from Scat; Mixed Scat). Data includes total seeds planted, total seeds germinated, proportion of seeds germinated and corresponding standard error of proportion at 30 days and 60 days after planting. Values were calculated using Microsoft Excel and are shown in Fig. 4.

| Treatment | Total seeds planted | 30 days after germination | | 60 days after germination | |
| --- | --- | --- | --- | --- | --- |
|  |  | Total germinated | Proportion germinated (mean +/- se) | Total germinated | Proportion germinated (mean +/- se) |
| Whole Berry | 2880 | 6 | 0.0021 (0.0001) | 60 | 0.0201 (0.0027) |
| Seeds from Berry | 432 | 123 | 0.2847 (0.0217) | 190 | 0.4398 (0.0239) |
| Seeds from Scat | 432 | 63 | 0.1458 (0.0170) | 135 | 0.3125 (0.0223) |
| Mixed Scat | 1295 | 155 | 0.1200 (0.0090) | 349 | 0.2693 (0.0123) |

**Table A3:** Result of generalized linear mixed model (alpha = 0.05) for 30 days and 60 days after planting, calculated using proportion of seeds germinated, weighted by number of seeds planted. Treatment Odds Ratios are relative to Whole Berry treatment.

|  | **proportion germinated** | | |
| --- | --- | --- | --- |
| *Predictors* | *Odds Ratios* | *CI* | *p* |
| (Intercept) | 0.00 | 0.00 – 0.00 | **<0.001** |
| [Seedsfromberry] | 280.95 | 146.46 – 538.95 | **<0.001** |
| [Seedsfromscat] | 116.84 | 33.84 – 403.45 | **<0.001** |
| [Mixedscat] | 56.72 | 16.41 – 196.04 | **<0.001** |
| days factor [60 days] | 3.62 | 3.04 – 4.31 | **<0.001** |
| **Random Effects** | | | |
| σ^2^ | 3.29 | | |
| τ_00_ _jiffy:id_ | 1.97 | | |
| τ_00_ _id_ | 0.57 | | |
| ICC | 0.44 | | |
| N _jiffy_ | 432 | | |
| N _id_ | 9 | | |
| Observations | 864 | | |
| Marginal R^2^ / Conditional R^2^ | 0.466 / 0.699 | | |
