# Supplementary material for: Effects of bear endozoochory on germination and dispersal of huckleberry in the Canadian Rocky Mountains
Source: PLoS One. 2024 Nov 6;19(11):e0311809. doi: 10.1371/journal.pone.0311809 (PMC11540177; doi:10.1371/journal.pone.0311809)
Supplement: Attachment — Submitted filename: PLOS ONE_review.pdf [file pone.0311809.s002.pdf]

REVIEW OF THE MANUSCRIPT

“Effects Of Bear Endozoochory On Germination And Dispersal Of Huckleberry In The Canadian Rocky Mountains” (PONE-D-24-27272)

Submitted for publication in PLOS ONE

This study investigates the role of bears in the dispersal and germination of huckleberry (*Vaccinium* spp.) seeds in the Canadian Rocky Mountains. The authors performed ex-situ germination experiments under controlled conditions to assess the effects of bear ingestion on huckleberry seed germination, distinguishing four different germination treatments. These treatments allowed them to test the effects of deinhibition, scarification, and fertilization promoted by bear ingestion and defecation on *Vaccinium* seed germination rates. The authors found that deinhibition had the strongest positive effect on seed germination rates, while the other two processes promoted by bear ingestion and defecation (scarification and fertilization) had neutral to moderately negative effects. Additionally, the authors combined published data on bear gut retention times and bear movement from radio-collared grizzly bears to calculate the potential distances at which grizzly bears can disperse the seeds in the Canadian Rocky Mountains. They estimated that the average distance at which bears disperse the seeds exceeds one kilometer and that the potential area of seed dispersal may be up to 150 km^2. Based on their results, the authors highlight the importance of the ecosystem services provided by bears in the region.

In general, the manuscript is well-written and easy to follow, and the presentation of results is clear. However, I believe the overall quality may still improve with some substantial modifications. The flow of some parts of the document, especially the introduction and the discussion, can be improved by implementing several changes not only in the wording but also in the overall structure. Additionally, I have some concerns about the assumptions made regarding the germination treatments, which makes me wonder about the actual ecological implications of the results obtained. Below, I provide a detailed list of comments and suggestions for each section of the manuscript.

ABSTRACT:

My main suggestion for the abstract is to emphasize more the main goal of the study. The authors mention the three processes that may affect seed germination rates after being ingested and defecated by frugivores (i.e., deinhibition, scarification, and fertilization). However, the authors do not clearly specify in the abstract that the study's goal is to isolate the effects of these three processes to describe their individual effects on the fate of the defecated seeds. Additionally, experimental treatments must be briefly described in the abstract. Even if it seems obvious to the authors, the link between the different treatments and the processes should be explicitly described so potential readers can understand why these four treatments were chosen.

- L27: Replace “influence” with “benefit.”
- L39: Modify to “can increase germination in huckleberry seeds.”

- L39-42: This sentence needs rewriting. It is more precise to say that around 50% of the seeds defecated by bears in the region are dispersed more than 1 km away from feeding places (and up to 7 km) and that the seed dispersal area can cover up to 150 km^2.
- L41: The definition of GRT50 is imprecise. The authors refer to the time when approximately half of the animals would have defecated, but the concept actually denotes the time when 50% of the cumulative weight of fecal remains had been defecated after the ingestion of specific food items (from Elfström et al. 2013).
- L42: As the seed shadow does not only include the surface but also the spatial distribution of seeds within this surface (i.e., different seed densities according to the distance to the feeding place), I recommend rephrasing "the resulting seed shadow" to "the surface covered by the seed shadow."
- L43: The effectiveness of the seed dispersal services provided by a disperser agent is traditionally evaluated based on the quantity and quality of the seeds dispersed, not based on the seed shadow covered by its dispersal services.
- L45-47: The fact that bear-berry interactions can benefit both bears and berry species is independent of increasing levels of human use and climate change. What is relevant as a final message is that these increasing pressures may threaten the ecosystem services provided by bears, with potential consequences not only for Vaccinium species but also for the entire ecosystem.

KEW WORDS: Some key words are already included in the title (bear, endozoochory, huckleberry). Please, replace them by others that are not.

INTRODUCTION:

The entire introduction would benefit from some changes in the order in which the main ideas are presented. For instance, instead of starting by discussing the bear-berry relationship (the first two paragraphs in the current format are about *Vaccinium* species and the importance of berries for bears), the authors must consider starting by introducing general ecological concepts such as frugivory and seed dispersal, highlighting their vital role in ecosystem functioning. From that, the authors may narrow down the focus, concluding with a final paragraph where they describe their study system and the main objectives of this work. A possible order for a more general introduction could look as follows:

- Paragraph 1: Frugivory and seed dispersal: concepts and main benefits for both animals and plants.
- Paragraph 2: Seed dispersal effectiveness: the need to assess germination as animal ingestion directly affects the qualitative component of seed dispersal services.
- Paragraph 3: Potential determinants of seed dispersal effectiveness traditionally neglected in seed dispersal studies: the importance of long-distance dispersal events, benefits for the plants, and the unique role of large frugivores in providing such services.
- Paragraph 4: Brief description of the study system (just a couple of sentences about the relevance of bear-berry interactions) and main objectives of the study.

Then, I would move the specific paragraphs about *Vaccinium* species (the first paragraph in the current version) and the importance of fruits and particularly berries in the bear diet (the second paragraph) to the methods section, where the authors may add a section called "Study Species," including two

subsections: one about the main characteristics of *Vaccinium* species and another about bears and the importance of fleshy fruits for these species.

Additionally, I personally miss a deeper description of the three processes potentially affecting seed germination during the ingestion and defecation by animals. For instance, do the authors know about any specific compound promoting deinhibition? Is scarification affected by seed size and seed species? Are the authors aware of any published information about the possible role of scat remains as seed fertilizer?

- L75-77: The last sentence of this paragraph is not accurate. There is a vast amount of literature about this mutualistic interaction. As most of these studies have been conducted either in the tropics or have focused on birds, I recommend the authors to rephrase this sentence to highlight that studies about the role of large frugivores in temperate ecosystems have often been neglected.
- L78-79: Replace with "differs according to the animal and plant species involved."
- L83: "it is only <u>one</u> of several…"
- L84-87: The text flow will improve if the authors delete the part about the whole berry treatment. Doing so will directly link the part about the three processes by which endozoochory may affect germination with the previous sentence stating that scarification is only one of these processes.
- L93-94: Please remove the part about the seed treatments from the introduction and explain them in more detail in the methods.
- L110: Greater importance in what context? I would just say that large frugivores contribute most to the long-distance dispersal events.
- L109-111: As written, it seems that Lalleroni *et al.* compared seed dispersal distances by different taxa, which is not true. Please rephrase or add extra references that support this comparison.
- L113: "and dispersal <u>distances</u> of huckleberry…"

MATERIAL AND METHODS:

My major concern about this section is regarding some assumptions made by the authors to calculate the germination rates of the different treatments.

In the whole berry treatment, the authors used 5 *Vaccinium* fruits to estimate the average number of seeds per berry. Regardless of the small sample size (only 5 fruits), which I consider insufficient to provide a robust estimation of the mean number of seeds per berry, I have two doubts that I think are not properly clarified in the manuscript:

1. Since the authors assigned all *Vaccinium* species as huckleberry (L53), they do not provide any information about the species collected. Did all the collected fruits (including those used for seed counting and those used for the germination trial) belong to the same *Vaccinium* species? Which one? This is important because different *Vaccinium* species may have different average numbers of seeds per fruit.
2. The authors collected huckleberry fruits around each of the bear scats collected. However, they do not provide any information about the original location of the 5 fruits collected, nor about the criteria followed to choose the fruits (the only thing mentioned is that they collected ripe fruits). For instance, seed set per *Vaccinium myrtillus* fruit is highly variable, potentially affected by environmental variables, and with heavier fruits containing on average larger numbers of seeds[1-4]. If the authors did

not control for fruit size and other factors potentially affecting seed production, then I would assume a significant variability in seed numbers, which could severely compromise the accuracy of the results obtained.

1-García-Rodríguez A., Albrecht J., Frydryszak D., Parres A., & Selva N. (2024). Interactive effects of elevation and canopy affect bilberry performance in a temperate coniferous region. Plant Ecology, 225(2), 81-91.
2-Pato, J., & Obeso, J. R. (2012). Growth and reproductive performance in bilberry (Vaccinium myrtillus) along an elevation gradient. Ecoscience, 19(1), 59-68.
3-Ranwala, S. M., & Naylor, R. E. (2004). Production, survival and germination of bilberry (Vaccinium myrtillus L.) seeds. Botanical Journal of Scotland, 56(1), 55-63.
4-Selås, V. (2000). Seed production of a masting dwarf shrub, Vaccinium myrtillus, in relation to previous reproduction and weather. Canadian Journal of Botany, 78(4), 423-429.

Similarly, I have serious concerns about the accuracy of the results of the mixed scat treatment. In L198-199, the authors explain that they planted about 12 seeds along with a berry-sized pinch of scat. However, in L404-409, the authors admit that this procedure may have led to differences in the number of seeds per pellet, potentially compromising the results obtained. As they aimed at having very similar (or even identical) seed numbers in all the mixed scat treatment pellets, I would have recommended the authors that, instead of selecting "berry-sized" pinches of different scats, they could simply mix/homogenize a unique bear scat in order to have similar seed densities within different portion of the scat, and then add a standardized subsample from this scat (for instance 0.5 grams) to each pellet.

Additionally, since the authors planted different amounts of seeds per pellet in each treatment (~27 seeds per pellet in the whole berry treatment, ~12 in the mixed scat treatment, and 4 in the seeds from berry and seeds from scat treatments), possible effects of competition among seeds may have been masked by the effects of the different treatments. For instance, *Vaccinium myrtillus* seed germination rates are negatively affected by the number of seeds planted together, suggesting that competition among seeds is stronger with increasing seed densities[5]. In order to discard competition effects, the authors must have planted similar seed numbers in the 4 treatments considered, instead of planting different seed numbers in each treatment.

5- García-Rodríguez, A., Albrecht, J., Farwig, N., Frydryszak, D., Parres, A., Schabo, D. G., & Selva, N. (2022). Functional complementarity of seed dispersal services provided by birds and mammals in an alpine ecosystem. *Journal of Ecology*, *110*(1), 232-247.

- L146: "scats."
- L157 (Figure 1): Did the authors collect fruits from each of the six sites where they collected bear scats? I see seven sites in the figure, but the authors mention collecting only six bear scats containing huckleberry seeds.
- L231: See my comment above about the definition of GRT50.
- L249-254: Information about the family distribution and the link function used is missing for the GLMM.
- L254: "repeated measures" instead of "repeat measures"? Additionally, I do not understand why the authors used each Jiffy pellet as a random intercept. I think that using each tray as a random intercept would have been a better option, allowing control for possible differences in germination rates according to the tray.

RESULTS:

- L257-258: the current sentence may result confusing because both Figure 4 and Table 3 show differences in germination rates between measurements at day 30 and day 60 after planting. I would rather specify that differences among treatments were consistent across time.
- L259: to avoid confusion, replace “samples” with “seeds”.
- L262: to avoid subjectivity, replace “moderate germination” with “intermediate germination rates”.
- L264-278: I think this entire paragraph needs rewriting. It mixes actual results (differences among treatments) with possible explanations of these results (the role of the different processes - deinhibition, scarification, fertilization- in the results, the discussion about which treatments naturally occur in nature, etc.). The latter must be included in the discussion, not in the results.
- L295: “Most germination happened within the first two months after planting” implies that some germination still occurred afterwards, but according to the authors, they only checked the first two months (L172-173). Did the authors check germination beyond these two months after planting? If not, they must explain in the methods why they chose two months as the length limit for the germination trial (e.g., similar studies using the same period, N days without any germination reported, etc.).

DISCUSSION:

The discussion can be substantially improved by making several changes. For instance, some parts of the discussion in the current version of the manuscript look like a repetition of the introduction and the results. In this sense, The authors must highlight the importance of their findings and discuss them in the context of previous knowledge, avoiding presenting the results in detail again (for instance, for L310-319 I think it would be sufficient to state that the study demonstrates that bear ingestion promotes huckleberry seed germination and suggests that scarification have a more significant and positive effect than deinhibition and fertilization). The integration of these findings into the overall discussion can also be substantially improved (for instance, sometimes the authors repeat some results and then mention previous publications with similar findings, but how the present study adds new knowledge to the existing literature must be better developed). In addition, I recommend the authors to elaborate more some aspects that are poorly addressed in the discussion, such as whether they think or not that the election of only one plant species may have affected the results obtained in the germination trials. I also miss a deeper explanation of the possible reasons behind the results obtained (e.g., the reasons why deinhibition likely plays a more relevant role than fertilization and scarification).

- L307-310: Please, delete this part from the discussion. This information is already provided in the introduction. Instead, start the discussion highlighting the most important findings of the study, but without repeating the results in detail (e.g. “this study demonstrates that bear ingestion facilitates huckleberry seed germination, suggesting that scarification is the main process involved. In addition, this study confirms that grizzly bears may disperse a significant proportion of the seeds they consume over distances greater than 1 km away from the feeding areas”).
- L315-316: Why do the authors think that fertilization may be more important for seedling survival than for germination? Do they have any data or references to support this suggestion?
- L322-327: The references cited to support the heterogeneous effects of scarification on seed germination rates use different fruit species with very different seed sizes. The authors make a too-general assumption when they state that deinhibition have a greater positive effect on germination than scarification without considering that scarification effects may vary according to seed size. For

Vaccinium species, their seeds are very small, and it is likely that animal digestion does not produce any scarification. I strongly recommend the authors to at least acknowledge that seed size is likely to be an essential factor explaining the different effects of scarification on seed germination rates of different species. In this sense, it is more interesting to compare their results with other studies analyzing differences in germination rates of *Vaccinium* seeds under different treatments, for instance:

  - Honkavaara, J., Siitari, H., Saloranta, V., & Viitala, J. (2007, January). Avian seed ingestion changes germination patterns of bilberry, Vaccinium myrtillus. In *Annales Botanici Fennici* (pp. 8-17). Finnish Zoological and Botanical Publishing Board.
  - García-Rodríguez, A., Albrecht, J., Szczutkowska, S., Valido, A., Farwig, N., & Selva, N. (2021). The role of the brown bear Ursus arctos as a legitimate megafaunal seed disperser. *Scientific reports*, *11*(1), 1282.
  - Steyaert, S. M., Hertel, A. G., & Swenson, J. E. (2019). Endozoochory by brown bears stimulates germination in bilberry. *Wildlife Biology*, *2019*(1), 1-5.
  - Traveset, A., & Willson, M. F. (1997). Effect of birds and bears on seed germination of fleshy-fruited plants in temperate rainforests of southeast Alaska. *Oikos*, 89-95.

- L350: see my comment above about the definition of GRT50.
- L352-363: I recommend the authors to discuss their results about seed dispersal distances in the context of previous studies conducted about the role of bears as long-distance seed dispersers and emphasize this vital role in the current context of absence or scarcity of other large frugivores in temperate and boreal regions. The uniqueness of bears as long-distance seed dispersers has been studied not only for brown bears, but also for other bear species such as the Asiatic black bear. Some references that could be valuable contributions to improve the discussion:
  - Lalleroni, Aurélie, et al. "Exploring the potential of brown bear (Ursus arctos arctos) as a long-distance seed disperser: a pilot study in South-Western Europe." Mammalia 81.1 (2017): 1-9.
  - Koike, S., Masaki, T., Nemoto, Y., Kozakai, C., Yamazaki, K., Kasai, S., ... & Kaji, K. (2011). Estimate of the seed shadow created by the Asiatic black bear Ursus thibetanus and its characteristics as a seed disperser in Japanese cool-temperate forest. Oikos, 120(2), 280-290.
  - Tsuji, Y., Okumura, T., Kitahara, M., & Jiang, Z. (2016). Estimated seed shadow generated by Japanese martens (Martes melampus): comparison with forest-dwelling animals in Japan. Zoological science, 33(4), 352-357.
  - Tsunamoto, Y., et al. "Timing of fruiting rather than topography determines the direction of vertical seed dispersal by mammals and birds." bioRxiv (2020): 2020-05.
- L381-386: recent studies have already suggested that mammals, and particularly bears, may help plant species cope with climate change by dispersing their seeds uphill. Please, use the existing literature to support this part of the discussion. Some references that may be helpful:
  - González-Varo, J. P., López-Bao, J. V., & Guitián, J. (2017). Seed dispersers help plants to escape global warming. *Oikos*, *126*(11), 1600-1606.
  - Naoe, S., Tayasu, I., Sakai, Y., Masaki, T., Kobayashi, K., Nakajima, A., ... & Koike, S. (2016). Mountain-climbing bears protect cherry species from global warming through vertical seed dispersal. *Current Biology*, *26*(8), R315-R316.
- L395-403: This is the first mention of mortality numbers in the study. I recommend the authors to mention in the methods that they checked mortality of the germinated seedlings (explaining in detail how this monitoring was performed) and report the outcomes of this monitoring in the results. In addition to this, I recommend to move this part of the discussion to the first section of the discussion, which is about the germination trials.

LITERATURE CITED: some references have DOI, others do not. Furthermore, homogenize the style to fit journal requirements.

APPENDIX 1 – TABLE 1: most probably a visualization error due to the creation of the final PDF while submitting the manuscript, but there are several typos in the table.

TABLE 2: I would combine upper and lower tables for better visualization of results, for instance:

		30 days after germination		60 days after germination	
	Total seeds planted	Total germinated	Proportion germinated (mean per pellet ± s.e)	Total germinated	Proportion germinated (mean per pellet ± s.e)
Treatment					

I hope the authors find my comments and suggestions constructive and useful to improve this manuscript.

Yours sincerely, Alberto García-Rodríguez
