# Supplementary material for: Effects of bear endozoochory on germination and dispersal of huckleberry in the Canadian Rocky Mountains
Source: PLoS One. 2024 Nov 6;19(11):e0311809. doi: 10.1371/journal.pone.0311809 (PMC11540177; doi:10.1371/journal.pone.0311809)
Supplement: Attachment — Submitted filename: Response to Reviewers.docx [file pone.0311809.s003.docx]

Reviewer's Responses to Questions

**Comments to the Author**

Reviewer #1: 

ABSTRACT:

My main suggestion for the abstract is to emphasize more the main goal of the study. The authors mention the three processes that may affect seed germination rates after being ingested and defecated by frugivores (i.e., deinhibition, scarification, and fertilization). However, the authors do not clearly specify in the abstract that the study's goal is to isolate the effects of these three processes to describe their individual effects on the fate of the defecated seeds. Additionally, experimental treatments must be briefly described in the abstract. Even if it seems obvious to the authors, the link between the different treatments and the processes should be explicitly described so potential readers can understand why these four treatments were chosen.

- Agreed. We have changed the pitch of the abstract to more concretely link to the goal of the study “We conducted a germination experiment to assess the ways each mechanism of bear endozoochory affects germination success of huckleberry (Vaccinium spp*.*) in the southern Canadian Rocky Mountains.”

L27: Replace “influence” with “benefit.”

- done

L39: Modify to “can increase germination in huckleberry seeds.”’

- done

L39-42: This sentence needs rewriting. It is more precise to say that around 50% of the seeds defecated by bears in the region are dispersed more than 1 km away from feeding places (and up to 7 km) and that the seed dispersal area can cover up to 150 km².

- done

L41: The definition of GRT50 is imprecise. The authors refer to the time when approximately half of the animals would have defecated, but the concept actually denotes the time when 50% of the cumulative weight of fecal remains had been defecated after the ingestion of specific food items (from Elfström et al. 2013).

-thank you, we have changed this throughout

L42: As the seed shadow does not only include the surface but also the spatial distribution of seeds within this surface (i.e., different seed densities according to the distance to the feeding place), I recommend rephrasing “the resulting seed shadow” to “the surface covered by the seed shadow.”

- done

L43: The effectiveness of the seed dispersal services provided by a disperser agent is traditionally evaluated based on the quantity and quality of the seeds dispersed, not based on the seed shadow covered by its dispersal services. L45-47: The fact that bear-berry interactions can benefit both bears and berry species is independent of increasing levels of human use and climate change. What is relevant as a final message is that these increasing pressures may threaten the ecosystem services provided by bears, with potential consequences not only for Vaccinium species but also for the entire ecosystem.

- edited to the following “Development, resource extraction, and climate change may disrupt the beneficial relationship between bears and huckleberries, where huckleberries help bears gain fat, and bears help spread huckleberry seeds—a process that may become increasingly important as climate change alters habitats.”

KEW WORDS: Some key words are already included in the title (bear, endozoochory, huckleberry). Please, replace them by others that are not.

- Removed, and replaced with “Ursus arctos, deinhibition, fertilization, vaccinium, scarification, seed dispersal”

INTRODUCTION:

The entire introduction would benefit from some changes in the order in which the main ideas are presented. For instance, instead of starting by discussing the bear-berry relationship (the first two paragraphs in the current format are about Vaccinium species and the importance of berries for bears), the authors must consider starting by introducing general ecological concepts such as frugivory and seed dispersal, highlighting their vital role in ecosystem functioning. From that, the authors may narrow down the focus, concluding with a final paragraph where they describe their study system and the main objectives of this work. A possible order for a more general introduction could look as follows:

Paragraph 1: Frugivory and seed dispersal: concepts and main benefits for both animals and plants.

Paragraph 2: Seed dispersal effectiveness: the need to assess germination as animal ingestion directly affects the qualitative component of seed dispersal services.

Paragraph 3: Potential determinants of seed dispersal effectiveness traditionally neglected in seed dispersal studies: the importance of long-distance dispersal events, benefits for the plants, and the unique role of large frugivores in providing such services.

Paragraph 4: Brief description of the study system (just a couple of sentences about the relevance of bear-berry interactions) and main objectives of the study. Then, I would move the specific paragraphs about Vaccinium species (the first paragraph in the current

version) and the importance of fruits and particularly berries in the bear diet (the second paragraph) to the methods section, where the authors may add a section called “Study Species,” including two subsections: one about the main characteristics of Vaccinium species and another about bears and the importance of fleshy fruits for these species.

Additionally, I personally miss a deeper description of the three processes potentially affecting seed germination during the ingestion and defecation by animals. For instance, do the authors know about any specific compound promoting deinhibition? Is scarification affected by seed size and seed species? Are the authors aware of any published information about the possible role of scat remains as seed fertilizer?

- We have now considerably reworked the introduction and added the suggested paragraphs.

L75-77: The last sentence of this paragraph is not accurate. There is a vast amount of literature about this mutualistic interaction. As most of these studies have been conducted either in the tropics or have focused on birds, I recommend the authors to rephrase this sentence to highlight that studies about the role of large frugivores in temperate ecosystems have often been neglected.

-	Added *“The extent to which endozoochory by large vertebrates provides dispersal and fitness benefits to fruiting shrubs in temperate ecosystems remains a poorly studied mutualistic interaction.”*

L78-79: Replace with “differs according to the animal and plant species involved.”

- done

L83: “it is only one of several…”

- done

L84-87: The text flow will improve if the authors delete the part about the whole berry treatment. Doing so will directly link the part about the three processes by which endozoochory may affect germination with the previous sentence stating that scarification is only one of these processes.

- done

L93-94: Please remove the part about the seed treatments from the introduction and explain them in more detail in the methods.

- done

L110: Greater importance in what context? I would just say that large frugivores contribute most to the long-distance dispersal events. L109-111: As written, it seems that Lalleroni et al. compared seed dispersal distances by different taxa, which is not true. Please rephrase or add extra references that support this comparison.

-	rephrased and added more citations “Larger species, such as bears, that exhibit long gut retention times and large travel distances could have greater importance in long distance seed dispersal than smaller species with more local ranges and shorter gut retention times (Lalleroni et al., 2017, Traveset and Willson 1997, Fukui 2003).”

L113: “and dispersal distances of huckleberry…”

- done

MATERIAL AND METHODS:

My major concern about this section is regarding some assumptions made by the authors to calculate the germination rates of the different treatments.

In the whole berry treatment, the authors used 5 Vaccinium fruits to estimate the average number of seeds per berry. Regardless of the small sample size (only 5 fruits), which I consider insufficient to provide a robust estimation of the mean number of seeds per berry, I have two doubts that I think are not properly clarified in the manuscript:

1. Since the authors assigned all Vaccinium species as huckleberry (L53), they do not provide any information about the species collected. Did all the collected fruits (including those used for seed counting and those used for the germination trial) belong to the same Vaccinium species? Which one?

This is important because different Vaccinium species may have different average numbers of seeds per fruit.

- helpful point, yes all were the same species and we were too generic in the past draft. We’ve now added the species name of *Vaccinium membranaceum into the manuscript*

2. The authors collected huckleberry fruits around each of the bear scats collected. However, they do not provide any information about the original location of the 5 fruits collected, nor about the criteria followed to choose the fruits (the only thing mentioned is that they collected ripe fruits). For instance, seed set per Vaccinium myrtillus fruit is highly variable, potentially affected by environmental variables, and with heavier fruits containing on average larger numbers of seeds1-4. If the authors didnot control for fruit size and other factors potentially affecting seed production, then I would assume a significant variability in seed numbers, which could severely compromise the accuracy of the results

obtained.

- From our 5 fruits we obtained a mean of 27.8 seeds/fruit, which is very similar the results of (Nowak and Crone 2012) who counted the seeds of 10 huckleberry fruits and obtained an average of (x~26.67, SD~10.40). While there is variation between fruits our average of 27.8 appears reasonable and consistent with estimates from the same species collected 100-300 km away from our site.

1-García-Rodríguez A., Albrecht J., Frydryszak D., Parres A., & Selva N. (2024). Interactive effects of elevation and canopy affect

bilberry performance in a temperate coniferous region. Plant Ecology, 225(2), 81-91.

2-Pato, J., & Obeso, J. R. (2012). Growth and reproductive performance in bilberry (Vaccinium myrtillus) along an elevation

gradient. Ecoscience, 19(1), 59-68.

3-Ranwala, S. M., & Naylor, R. E. (2004). Production, survival and germination of bilberry (Vaccinium myrtillus L.) seeds. Botanical

Journal of Scotland, 56(1), 55-63.

4-Selås, V. (2000). Seed production of a masting dwarf shrub, Vaccinium myrtillus, in relation to previous reproduction and

weather. Canadian Journal of Botany, 78(4), 423-429.

Similarly, I have serious concerns about the accuracy of the results of the mixed scat treatment.

In L198-199, the authors explain that they planted about 12 seeds along with a berry-sized pinch of scat. However, in L404-409, the authors admit that this procedure may have led to differences in the number of seeds per pellet, potentially compromising the results obtained. As they aimed at having very similar (or even identical) seed numbers in all the mixed scat treatment pellets, I would have recommended the authors that, instead of selecting “berry-sized” pinches of different scats, they could simply mix/homogenize a unique bear scat in order to have similar seed densities within different portion of the scat, and then add a standardized subsample from this scat (for instance 0.5 grams) to each pellet.

- This is a good idea and something we will consider trying in the future.

Additionally, since the authors planted different amounts of seeds per pellet in each treatment (~27 seeds per pellet in the whole berry treatment, ~12 in the mixed scat treatment, and 4 in the seeds from berry and seeds from scat treatments), possible effects of competition among seeds may have been masked by the effects of the different treatments. For instance, Vaccinium myrtillus seed germination rates are negatively affected by the number of seeds planted together, suggesting that competition among seeds is stronger with increasing seed densities5. In order to discard competition effects, the authors must have planted similar seed numbers in the 4 treatments considered, instead of planting different seed numbers

in each treatment.

5- García‐Rodríguez, A., Albrecht, J., Farwig, N., Frydryszak, D., Parres, A., Schabo, D. G., & Selva, N. (2022). Functional

complementarity of seed dispersal services provided by birds and mammals in an alpine ecosystem. Journal of Ecology, 110(1),232-247.

- This is a fair point. There are different ways to look at the issue I suppose. We were looking to replicate the real world effects of these different mechanisms and competition may interact and be part of the mechanism perhaps. I suppose we could’ve done all treatments to match the mean of the whole berry (27 seeds) to standardize the competition effect. Had we done that there was little difference between whole berry and mixed scat we would likely be wondering if the result was useful because we artificially increased the densities of seeds above the density we see distributed in scats. I think the best we can do here is be clear on our methods and add these thoughts into the discussion to guide future work. I think what we did is legitimate and worth publishing, but could be improved on in the future.
- We added the following to the discussion: *“Another source of potential error was the varying number of seeds in each Jiffy pellet. We attempted to controlled for the number of seeds planted in each treatment (Figure 2) by conducting our statistical tests on the proportion of seeds that germinated. We used the average number of seeds present in each Whole Berry and Mixed Scat treatment. It is likely that the actual number of seeds varied between individual Jiffy pellets in the these treatments, suggesting some level of unaccounted variation in our results. This level of variation is not expected to change the overall results, especially where the effect sizes are large such as the comparisons with Whole Berry. Similarly, the treatments could have been differentially affected by competition between seeds. There is evidence that germination rates for some Vaccinium species can be affected by seed density (García-Rodríguez et al. 2022). It would be wise for future works to match the number of seeds across trails, at least in a portion of the samples, to assess the effects of competition. On the other hand, differing density of seeds is actually part of the endozoochory process as we have shown here with the approximately 27 seeds within each berry, and only 12 seeds within a similar volume of scat. Thus, while controlling for competition in a portion of trials in future work would be wise, we also urge investigators match real world conditions in a portion of their treatments to allow for estimates of the expected effect size in nature.”*

- L146: “scats.”

- done

L157 (Figure 1): Did the authors collect fruits from each of the six sites where they collected bear scats? I see seven sites in the figure, but the authors mention collecting only six bear scats containing huckleberry seeds.

-The seven collection sites were in 4 general “areas” as can be seen by the clusters. At each of these 4 areas we collected:

1. Stormcat Flats: one scat site

2.  East side of Elk Valley above Fernie: one scat and one berry collection site

3.  Waterton Continental Divide: two scat and one berry sample site

4.  Sand Creek: two scats and one berry sample site

All this to say that 6 scats were collected at 4 different areas and berries were sampled from 3 of these locations. I think the 7 the reviewer is referring to is that on the map, some of these locations are so close together that they are indistinguishable, leading to 7 stars marking “sites”. Locations and what was collected at each site is presented in Table 1 in appendix.

L231: See my comment above about the definition of GRT50.

-Helpful comment. We’ve made our definition clearer, thank you. “The 5-7 hour period reflects the average GRT when bears had deposited 50% of their cumulative fecal weight following an initial feeding of berries (GRT50%), and the 12-18 hour period represents the maximum time for deposition (GRTmax) as per Elfström et al. (2013).”

L249-254: Information about the family distribution and the link function used is missing for the GLMM.

-added the following “We input the number of seeds that germinated as the response variable and a weighted the response by the number of seeds initially planted so that a proportion was effectively estimated using a binomial model with logit link. “

L254: “repeated measures” instead of “repeat measures”? Additionally, I do not understand why the authors used each Jiffy pellet as a random intercept. I think that using each tray as a random intercept would have been a better option, allowing control for possible differences in germination rates according to the tray.

-jiffy pellet is to account for the repeated measures of each jiffy pellet (30 and 60 days)

RESULTS:

L257-258: the current sentence may result confusing because both Figure 4 and Table 3 show

differences in germination rates between measurements at day 30 and day 60 after planting. I would rather specify that differences among treatments were consistent across time.

- Agreed, thanks, changed as suggested.

L259: to avoid confusion, replace “samples” with “seeds”.

-done

L262: to avoid subjectivity, replace “moderate germination” with “intermediate germination rates”.

-done

L264-278: I think this entire paragraph needs rewriting. It mixes actual results (differences among treatments) with possible explanations of these results (the role of the different processes

deinhibition, scarification, fertilization- in the results, the discussion about which treatments naturally occur in nature, etc.). The latter must be included in the discussion, not in the results.

- I moved a few things around here to improve flow. Respectfully, we believe this comment is a matter of personal preference. We have decided to retain the small bit of inference in the results as is.

L295: “Most germination happened within the first two months after planting” implies that some germination still occurred afterwards, but according to the authors, they only checked the first two months (L172-173). Did the authors check germination beyond these two months after planting? If not, they must explain in the methods why they chose two months as the length limit for the germination trial (e.g., similar studies using the same period, N days without any germination reported, etc.).

- Removed this sentence, it wasn’t needed. But added citations to how we chose our time period in methods. The Nowak and Crone 2012 work only used 1 month and we went for another month to ensure the results were the same. “The germination trial occurred over two months, from November 29^th^, 2021 until January 29^th^, 2022. Past studies looking at huckleberry germination have monitored germination during a four week period (Nowak and Crone 2012). We extended our trial to eight weeks which allowed us to assess the effect of time on observed differences in germination rates between treatments.”

DISCUSSION:

The discussion can be substantially improved by making several changes. For instance, some parts of the discussion in the current version of the manuscript look like a repetition of the introduction and the results. In this sense, The authors must highlight the importance of their findings and discuss them in the context of previous knowledge, avoiding presenting the results in detail again (for instance, for L310-319 I think it would be sufficient to state that the study demonstrates that bear ingestion promotes huckleberry seed germination and suggests that scarification have a more significant and positive effect than deinhibition and fertilization). The integration of these findings into the overall discussion can also be substantially improved (for instance, sometimes the authors repeat some results and then mention previous publications with similar findings, but how the present study adds new knowledge to the existing

literature must be better developed). In addition, I recommend the authors to elaborate more some aspects that are poorly addressed in the discussion, such as whether they think or not that the election of only one plant species may have affected the results obtained in the germination trials. I also miss a deeper explanation of the possible reasons behind the results obtained (e.g., the reasons why deinhibition likely plays a more relevant role than fertilization and scarification).

L307-310: Please, delete this part from the discussion. This information is already provided in the introduction. Instead, start the discussion highlighting the most important findings of the study, butwithout repeating the results in detail (e.g. “this study demonstrates that bear ingestion facilitates huckleberry seed germination, suggesting that scarification is the main process involved. In addition, this study confirms that grizzly bears may disperse a significant proportion of the seeds they consume over distances greater than 1 km away from the feeding areas”).

- Done, thank you for the suggestion

L315-316: Why do the authors think that fertilization may be more important for seedling survival than for germination? Do they have any data or references to support this suggestion?

- We have added a citation for this idea *“It is possible that fertilization effects would be more pronounced in seedling survival and growth rate rather than germination (Traveset et al. 2001).”*

L322-327: The references cited to support the heterogeneous effects of scarification on seed

germination rates use different fruit species with very different seed sizes. The authors make a too-general assumption when they state that deinhibition have a greater positive effect on germination than scarification without considering that scarification effects may vary according to seed size. For Vaccinium species, their seeds are very small, and it is likely that animal digestion does not produce any scarification. I strongly recommend the authors to at least acknowledge that seed size is likely to be an essential factor explaining the different effects of scarification on seed germination rates of different species. In this sense, it is more interesting to compare their results with other studies analyzing differences in germination rates of Vaccinium seeds under different treatments, for instance:

o Honkavaara, J., Siitari, H., Saloranta, V., & Viitala, J. (2007, January). Avian seed ingestion changes germination patterns of bilberry, Vaccinium myrtillus. In Annales Botanici Fennici (pp. 8-17). Finnish Zoological and Botanical Publishing

Board.

o García-Rodríguez, A., Albrecht, J., Szczutkowska, S., Valido, A., Farwig, N., & Selva, N. (2021). The role of the brown bear Ursus arctos as a legitimate megafaunal seed disperser. Scientific reports, 11(1), 1282.

o Steyaert, S. M., Hertel, A. G., & Swenson, J. E. (2019). Endozoochory by brown bears stimulates germination in bilberry. Wildlife Biology, 2019(1), 1-5.

o Traveset, A., & Willson, M. F. (1997). Effect of birds and bears on seed germination of fleshy-fruited plants in temperate rainforests of southeast Alaska. Oikos, 89-95.

- Thank you, this is a helpful critique, we have now added the following into the discussion: *“Among many factors, seed size is thought to play a dominant role in variation in the effect of scarification as the amount of pulp, thickness of the seed coat, and gut retention times vary between fruits with different seed sizes (Chang et al., 2012). Huckleberry seeds are small, especially relative to the amount of pulp around them, consistent with the effects observed in our work where the removal of this pulp (deinhibition) during digestion produced the largest benefits to germination. Indeed, this pattern has been observed in multiple Vaccinium species consumed by bears (Nowak and Crone 2012 and García-Rodríguez et al. 2021).”*

L350: see my comment above about the definition of GRT50.

-Thanks, fixed *“Studies of captive bears fed a diet of berries reported a gut retention time when half of the cumulative fecal weight was deposited (GRT_50%_) of approximately 6 h and 15 minutes (Elfström et al. 2013).”*

L352-363: I recommend the authors to discuss their results about seed dispersal distances in the

context of previous studies conducted about the role of bears as long-distance seed dispersers and

emphasize this vital role in the current context of absence or scarcity of other large frugivores in

temperate and boreal regions. The uniqueness of bears as long-distance seed dispersers has been

studied not only for brown bears, but also for other bear species such as the Asiatic black bear. Some references that could be valuable contributions to improve the discussion:

o Lalleroni, Aurélie, et al. "Exploring the potential of brown bear (Ursus arctos arctos) as a long-distance seed disperser:

a pilot study in South-Western Europe." Mammalia 81.1 (2017): 1-9.

o Koike, S., Masaki, T., Nemoto, Y., Kozakai, C., Yamazaki, K., Kasai, S., ... & Kaji, K. (2011). Estimate of the seed shadow

created by the Asiatic black bear Ursus thibetanus and its characteristics as a seed disperser in Japanese cool‐temperate

forest. Oikos, 120(2), 280-290.

o Tsuji, Y., Okumura, T., Kitahara, M., & Jiang, Z. (2016). Estimated seed shadow generated by Japanese martens (Martes

melampus): comparison with forest-dwelling animals in Japan. Zoological science, 33(4), 352-357.

o Tsunamoto, Y., et al. "Timing of fruiting rather than topography determines the direction of vertical seed dispersal by

mammals and birds." bioRxiv (2020): 2020-05.

- *Added the following to the discussion: “The spatial extent of grizzly bears seed dispersal estimated in our study is consistent with the results from studies across the world. For example, Lalleroni et al., (2017) assessed the seed dispersal potential for three brown bears (also Ursus arctos) in southwest Europe using the same GRT’s as our study. Their results closely mirror ours with the average GRT_50%_ between 1.09-1.34 km, similar to our estimate of 1.1 km. Both our study and that of Lalleroni et al. (2017) estimated dispersal distances 2-3 fold farther than Koike et al., (2011) who assessed Asiatic black bear (Ursus thibetanus) movements in Japan. Grizzly bears are known to be fruit consumers across their global range and their ability consumer large volumes of seeds and disperse them multiple kilometers highlights their important role as seed dispersers. Importantly, the relative role of each species in dispersing seeds must consider not only how far or how many seeds are dispersed, but also the density of the disperser on the landscape and the proportion of dispersed seeds that germinate and eventually turn into adult plants (García-Rodríguez et al. 2021c).”*

L381-386: recent studies have already suggested that mammals, and particularly bears, may help plant species cope with climate change by dispersing their seeds uphill. Please, use the existing literature to support this part of the discussion. Some references that may be helpful:

o González‐Varo, J. P., López‐Bao, J. V., & Guitián, J. (2017). Seed dispersers help plants to escape global

warming. Oikos, 126(11), 1600-1606.

o Naoe, S., Tayasu, I., Sakai, Y., Masaki, T., Kobayashi, K., Nakajima, A., ... & Koike, S. (2016). Mountain-climbing bears

protect cherry species from global warming through vertical seed dispersal. Current Biology, 26(8), R315-R316.

- Thanks, this is helpful. We have now added the following to the discussion *“Grizzly bear dispersal of huckleberry seeds in the Rocky Mountains is on the order of up to 6-11 km per feeding event and will support and likely exceed the minimum movement rate that huckleberry plants will need under climate change. Many other seed dispersers are also likely to support plant dispersal as climates change. For example, González‐Varo et al. (2017) show that pine marten (Martes martes) can disperse fruit seeds an average of 106 m upslopes, exceeding the 35 m/decade warming threshold expected under climate change. Naoe et al. (2016) show similar upslope movements of seeds by black bear. There is thus substantial evidence that multiple vertebrates consume, disperse, and support germination of fruit seeds and this process is likely to become even more important in abating deleterious effects of climate change on the distribution and population viability of fruiting species.”*

L395-403: This is the first mention of mortality numbers in the study. I recommend the authors to mention in the methods that they checked mortality of the germinated seedlings (explaining in detail how this monitoring was performed) and report the outcomes of this monitoring in the results. In addition to this, I recommend to move this part of the discussion to the first section of the discussion, which is about the germination trials.

-Added checking of mold to methods and results. Removed the seedling survival piece as we had no formal data on survival and added the following to discussion *“Mold was present on some Jiffy Pellets within the first two weeks of the trial, with the Mixed Scat treatment having the highest prevalence noted. If mould spores and/or propagules are present in bear scat, bears may also be dispersing mold alongside seeds. It is unknown if the presence of mold altered the germination of seeds. Thus this is a source of uncertainty in our results, a challenge which others have also faced in similar endozoochory trials Robertson et al. (2006).”*

LITERATURE CITED: some references have DOI, others do not. Furthermore, homogenize the style to fit journal requirements.

APPENDIX 1 – TABLE 1: most probably a visualization error due to the creation of the final PDF while submitting the manuscript, but there are several typos in the table.

- Must be a PDF creation as it looks OK on our end

TABLE 2: I would combine upper and lower tables for better visualization of results, for instance: 30 days after germination 60 days after germination

-Good idea, thanks. We have changed the table to reflect the structure proposed by the reviewer:

| Treatment | Total seeds planted | 30 days after germination | | 60 days after germination | |
| --- | --- | --- | --- | --- | --- |
|  |  | Total germinated | Proportion germinated (mean +/- se) | Total germinated | Proportion germinated (mean +/- se) |
| Whole Berry | 2880 | 6 | 0.0021 (0.0001) | 60 | 0.0201 (0.0027) |
| Seeds from Berry | 432 | 123 | 0.2847 (0.0217) | 190 | 0.4398 (0.0239) |
| Seeds from Scat | 432 | 63 | 0.1458 (0.0170) | 135 | 0.3125 (0.0223) |
| Mixed Scat | 1295 | 155 | 0.1200 (0.0090) | 349 | 0.2693 (0.0123) |

I hope the authors find my comments and suggestions constructive and useful to improve this manuscript.

Yours sincerely, Alberto García-Rodríguez

**Reviewer #2: Comments to the Authors**

MS. Ref. No.: PONE-D-24-27272
Title: " Effects of Bear Endozoochory on Germination and Dispersal of Huckleberry in the
Canadian Rocky Mountains” Kuijt et al.

This MS examines important aspects of seed and reproductive biology of huckleberry (Vaccinium sp.) through bear endozoochory in Canadian Rocky Mountains. In particular the authors investigated germination success (total germination percentage within 30 and 60 days in Jeffy pellets under controlled laboratory conditions) of four different seed treatments (whole berry, seeds from berry, seeds from scat and mixed scat) and effectively disentangled de-inhibition effect, scarification effect and fertilization effect. They demonstrated that probability of germination in huckleberry seeds in nature was strongly affected by the removal of inhibitory compounds through
pulp removal (de-inhibition effect), whereas scarification and fertilization did not. Additionally, they also studied contribution of bears in successfully dispersing seeds over long distances by combining literature and movement data.

In my opinion, the MS deserves to be published in PLOS ONE since it covers an interesting topic of scientific value and the results presented may provide new comprehension on it. Also, I believe that the authors have considered some specific concerns raised by recent literature (see Robertson et al. 2006, but also Rogers et al. 2021, which has not been cited in References and could be added and be considered in Discussion), where it has been highlighted the need to include intact fruits in germination test for investigation of frugivory impact on seed dispersal.

Also, some minor points that the authors need to reconsider before publication are the following:

Minor points

Line 31: avoid italics in “spp.”

- done

Lines 75 and 97-98: check and correct the style in “García-Rodríguez et al. 2021a,b” and “García-Rodríguez et al. 2022

- done

Lines 84, 114 and 121: correct author citation in “Robertson”

- done

Lines 92 and 116: in citation of Robertson et al., correct year in “2006”

- done

Line 313: correct in “scarification”

- done

Line 189-190: give information on the final volume of rehydrated Jeffy pellet. Also, the authors could add here more information why they choose to use peat substrate for germination trial and if this substrate simulate or not natural conditions where berries fall from plants.

- added the following “The 2 cm radius Jiffy pellets typically rehydrated to ~5 cm high, or a volume of ~75 cm^2^.” And “Jiffy pellets offer a consistent and controlled environment for germination trials, minimizing variability in soil composition, moisture retention, and nutrient availability. This is crucial for ensuring that observed differences in germination rates are due to the seeds themselves and not variations in the substrate. While the natural substrate that huckleberries would typically encounter in the forests and subalpine of southeast British Columbia is more complex and consists of well-drained soils, rich in organic matter, peat substrates do offer some similarities, particularly in terms of organic content and moisture retention.”

Line 206-207: the sentence is correct only for the Seeds from Berry and Seeds from Scat treatments. Add correct information for the other two treatments. Regarding this point: I wonder whether the comparison among Whole Berry and Seeds from Berry should be performed taking in account that in the same volume of each Jeffy pellet the authors tested germination of different numbers of seeds (27 vs. 4 seeds). Maybe it could be useful, if possible, to add an additional test to check if germination in Seeds from Berry Treatment does not change if 4 or 27 seeds were planted in one single Jeffy pellets. The same concern could be valid for Mixed Scat Treatment.

-Agreed. Removed the #4 and referenced Fig 2 which has the seed counts by treatment.

Lines 257-258: have the authors checked at the end of germination trial if the not germinated seeds in Whole Berry treatment were still viable or dead? I believe this information is very important to understand if the seeds are effectively dormant and not germinating because of deinhibitory effects from pulp or they died for lack of oxygen or fungal infection during test.
This aspect was also evidenced in Robertson et al. 2006, which could render more difficult to assess the impacts of endozoochory.

-Added checking of mold to methods and results. Removed the seedling survival piece as we had no formal data on survival and added the following to discussion *“Mold was present on some Jiffy Pellets within the first two weeks of the trial, with the Mixed Scat treatment having the highest prevalence noted. If mould spores and/or propagules are present in bear scat, bears may also be dispersing mold alongside seeds. It is unknown if the presence of mold altered the germination of seeds. Thus this is a source of uncertainty in our results, a challenge which others have also faced in similar endozoochory trials Robertson et al. (2006).”*
